# Teachers’ Well-Being Forced to Work from Home Due to COVID-19 Pandemic: Work Passion as a Mediator

**DOI:** 10.3390/ijerph192215095

**Published:** 2022-11-16

**Authors:** Elżbieta Kasprzak, Karolina Mudło-Głagolska

**Affiliations:** Faculty of Psychology, Kazimierz Wielki University, 1 Staff St, 85-064 Bydgoszcz, Poland

**Keywords:** well-being, affect, engagement, job crafting, work passion, teachers, working from home, COVID-19 pandemic

## Abstract

Background: This study examines the relationship between perceived demands (workload and organizational constraints) of teachers’ work during the online period of schooling during the COVID-19 crisis and well-being (emotions, engagement, and job crafting), with work passion as a mediator. Methods: The survey was carried out on a sample of 383 teachers during the first wave of the COVID-19 pandemic. The Scale of Organizational Constraints and the Workload Scale, the Passion Scale adapted for work, the Job Crafting Questionnaire, the Utrecht Scale of Work Engagement, and the Scale of Positive and Negative Experience was used. Results: Harmonious passion strengthened the positive relationships between workload and organizational constraints and job crafting and weakened the negative relationship with positive emotions and the positive one with negative emotions. The positive relationship between workload and engagement has been strengthened by harmonious passion. The negative relationship between organizational constraints and engagement became positive and weaker. Relationships between variables were weakened, i.e., workload and engagement, organizational constraints and job crafting, or strengthened, i.e., organizational constraints and engagement, by an obsessive passion. In tested models, obsessive passion has the opposite effect and is weaker than harmonious passion. Conclusion: The structural equation modeling (SEM) confirmed that work passion, mainly harmonious, is a mechanism explaining the relationship between the demands of forced work from home with teachers’ well-being.

## 1. Introduction

Due to the COVID-19 pandemic, remote work ceased to be an optional form reserved for well-earning knowledge workers and became a common (or only) way of working in many professions. Over one year in the EU, the number of remote workers more than doubled from 5.4% in 2019 to 12% in 2020 [1], and among adult Europeans with Internet access in July 2020, nearly 50% of employees worked from home (*N* = 91,753) [2]. The professional group was forced to work from home suddenly, and a masse were teachers —over 80% of European teachers in Spring 2020 started teaching online [2]. Teachers were left without sufficient organizational and technological support, and without effective communication with their headmasters and colleagues struggling with the same pandemic stress factors. The teachers’ workload has increased due to the need to learn and test online tools useful in education and to adapt the teaching materials to distance learning [3]. Teaching without dedicated remote work applications and sufficient ICT knowledge [4], in social isolation, they may perceive the school as an organization insufficiently supporting or limiting their work. Lizana and Vega-Fernandez proved that teachers working remotely during a pandemic feel more workload and health deterioration [5]. Pöysä et al. [6], who referred to the research of Bermejo-Toro et al. [7], emphasized that the average level of teacher vigor and dedication during COVID-19 was slightly higher than before. Moreover, recent studies give insight into mechanisms explaining teachers’ well-being in forced remote work. The effect of teachers’ stress on well-being is mediated by job crafting for people that score low on problem-focused coping [8]. On the other hand, Gao et al. [9] confirmed the problem-focused coping strategy as a direct mechanism for maintaining good health and reducing the risk of developing PTSD in the face of stress.

### 1.1. Teachers’ Well-Being and Its Determinants

Well-being is the state that makes a person happy and healthy and enables him to thrive and develop. In classical psychological models, well-being is described by subjective experiences (e.g., affect) and assessment (satisfaction) of different aspects of life or various life domains, e.g., work [10]. In more recent literature, one can find a broader picture of well-being, as all the affects, behaviors, attitudes, and cognitive processes that enable the thriving and growth of a person, as opposite to ill-being, including affective, behavioral, cognitive, and attitudinal factors that increase stress, weaken health and happiness [11]. Given the more burdensome conditions for teachers working remotely, this article focuses on well-being at work, especially on affective well-being, engagement as attitudinal well-being, and job crafting as behavioral well-being.

In this study, it is accepted that such a broad definition of well-being can be sustained or increased by positive mood, engagement, and productivity at work, like job crafting. Positive emotions, engagement, and proactivity are the components of wellbeing, such as in the PERMA model [12] or the psychological well-being model [13]. Moreover, engagement is defined explicitly as a form of well-being [14].

Affective well-being is an aspect of subjective well-being and includes emotions and moods [15]. Emotional reaction is a relevant and a sensitive indicator of the person’s attitude to the event, especially when the event is new and unexpected [16]. Emotion acts as information about new pandemic living and working conditions and prepares a person for adaptive behavior. Forced remote work and anxiety or uncertainty due to a pandemic risk are factors of the emotions and moods of employees.

Engagement reflects a person’s basic attitude to the job and is defined as “a positive, rewarding, work-related state of mind” [17] that is characterized by (1) vigor (i.e., energy, mental resilience, the willingness to invest effort in work, persistence also in the face of difficulties), (2) dedication (i.e., a sense of meaning, enthusiasm, inspiration, pride, and challenge); and (3) absorption (i.e., fully focusing and engrossing one’s work, a sense of quickly time passing and difficulties with detaching from work) [17]. Engagement fluctuates over time because it is the response to situational changes [18].

Job crafting is a proactive behavior initiated by an employee consisting in introducing changes in the performance of tasks, crafting relationships with co-workers, and thinking about work. Job crafting means expanding work boundaries, improving work in one’s position, maintaining interest and engagement at work, and seeking satisfaction and meaning in work [19]. In accordance with the JD-R model [20] and the job crafting model [19], behavioral job crafting is undertaken when an employee treats demands as challenges and, without the guidance of his superiors, independently changes work methods or social relations or resigns from ineffective activities (optimization of demands) [21,22]. Job crafting is undertaken thanks to professional resources, such as autonomy [19] and a proactive personality that supports a proactive strategy in adaptation processes [23]. The results of the study by Ingusci et al. suggest that high levels of workload increase the willingness to job craft [4]. Job crafting can represent an offensive strategy triggered by significant demands [24,25] but directed at seeking resources at work [26].

Job crafting enhances engagement [27], and engaged employees more often craft their work, and that way, employees feel spirals of profits of job resources and personal growth [26]. In previous research, job crafting in the form of seeking resources or challenges indirectly influenced engagement and satisfaction [27,28]. In our model, we consider job crafting (as well as affect and engagement) as a result of an overload of remote work mediated by a passion for work.

Coping with the demands at work is based on reactive or /and proactive actions, and these activities are accompanied by negative and/or positive emotions. The employee chooses reactive behavior when he or she prefers reducing demands and resource protection, i.e., narrowing the work boundaries, which means increased engagement in work and does not meet the criteria of job crafting.

### 1.2. Passion as a Mediator between Workload and Organizational Constraints and Well-Being

It is believed that passion for a teacher’s work is the factor that buffers the negative effect of demands on engagement, job crafting, and emotions. According to the Dualistic Model of Passion, passion includes the tendency to engage in pleasurable, personally important activities that take time and are part of identity [29]. Two dimensions of passion, harmonious passion (HP) and obsessive passion (OP), develop depending on the level of internalization of values, rewards, goals, and norms [30]. Both forms of passion are only relatively orthogonal (hence intraindividual both are active), although usually one of them is dominant.

Passion has mainly a motivational potential that provides energy to act [11,31]. Depending on the type, work passion strengthens or weakens job crafting and affect, and, regardless of the type, motivates engagement (although with different power in individual aspects of engagement).

Harmonious passion is a state inducing an activity that enhances affective, cognitive, attitudinal, and behavioral well-being [11]. Affective well-being, including emotions and moods [15], accompanies activities completed with passion, e.g., with work passion [29].

Moreover, harmonious passion is positively linked with job satisfaction and citizenship behavior at work [32], flow [33], engagement [31], hedonic and eudaimonic well-being, and awakening to purpose [34] and negatively links with burnout and intention to leave the organization [35,36,37]. Harmonious passion, directing the person’s attention to personally important activities and own resources, supports perceiving demands in forced work as a challenge and enhance the motivating role of demands. Harmonious passion enables employees to perform tasks flexibly, so they are more likely to focus better and discover what and how to improve at work to increase the sense of work, use their competencies, and facilitate the performance of tasks. Such activity of harmonious passion leads to positive outcomes, e.g., positive affect, absorption, and flow [38]. A person with harmonious passion is happy to craft and engage in his or her work.

Obsessive, uncontrolled passion, undertaken conditionally and poorly internalized in the employee’s identity, protects the ego; therefore, as a rule, it reduces or slightly increases engagement in work and positively explains burnout [32,36]. This suggests that job demands may enhance employees’ motivation through obsessive passion, forcing them to carry out their tasks in a rigid and maladaptive way to complete duties and maintain self-esteem. Moreover, people with obsessive passion cannot cut off work, turn off their emotions and stop thinking about work themselves [39]. Such response to job demands can generate emotional costs [36,37] and is not conducive to resource conservation [40].

Researchers confirmed that self-efficacy protects against the negative effects of emotional demands at work on engagement [41] or that self-efficacy and optimism are positively associated with flourishing when job demands are low and with engagement when demands (difficulties) are high [42]. We expect that passion for work will operate analogically. A harmonious passion for work enhances a person-job fit and leads to higher job satisfaction and a higher sense of control over the work environment. It is, therefore, likely that the teachers who work with passion will experience better well-being, and the passionate teachers who are tired regulate their energy and sustain their well–being.

However, it is believed that obsessive work passion induces self-defense behavior directed to limit demands and emotional tension and weakly or not at all strengthens engagement and job crafting, although, workload and organizational constraints generally motivate [23]. Additionally, obsessive passion mediates between workload and organizational constraints and negative affect due to following external rewards (e.g., social) that are hardly available in times of pandemic stress.

Moreover, it is supposed that the type of demands is important in predicting well-being, especially job crafting. The workload takes away energy, which decreases emotional well-being, engagement, and job crafting but this unfavorable relationship can be buffered by passion (harmonious and partially by obsessive passion). Organizational constraints perceived as a challenge encourage proactive behavior. People with harmonious passion are oriented toward curiosity, enjoyment, and challenges at work and are ready to craft their job. Employees with a defensive attitude towards work inherent in the obsessive passion struggle with the downsizing of demands and are not looking for resources such as proactive changes in work. So, we expect harmonious passion to mediate more strongly between organizational constraints and job crafting than obsessive passion.

To summarize, we assume that harmonious passion suppresses the negative correlation between all demands and emotions, engagement, and job crafting, whereas obsessive passion mediates negative affect and suppresses positive affect and (slightly) engagement. Thus, harmonious and obsessive passion enhance engagement regardless of the type of demands.

### 1.3. Present Study

The present study focuses on analyzing the mechanism that facilitates the psychological adaptation of teachers to stressful remote work in the COVID-19 context. Passion is an important subjective resource that can help adapt and sustain well-being in stressful work conditions. Hypothetical general models concern the relationship between the perception of workload and organizational constraints and well-being: affect, engagement, and job crafting, considering the mediating role of work passion. The appropriate theoretical background of this study is the Job Demands-Resources theory (JD-R) [26] and the dualistic model of passion [29].

The JD-R theory [26] describes work conditions as job demands and job resources. Job demands are understood as physical, psychological, social, and organizational aspects of the jobs that require physical and/or psychological effort and therefore are associated with physiological and/or psychological costs for employees. Job resources include all psychological, physical, and social organizational characteristics of work that facilitate goal achievement, reduce job demands and costs and increase learning, growth, and thriving. Demands at work take two forms, challenge or hindrance to job demand depending on its positive or negative outcomes, the context of job characteristics appraisal, individual interpretation of demands [43], and the number of significant demands at work [44]. Generally, demands are better predictors of health at work (i.e., burnout, length of absences, and depression) and job resources (and challenge job demands) better explain motivational processes at work (i.e., engagement–disengagement). The essence of job demands is that they use energy because they need to be met. Work resources initiate motivation (i.e., voluntarily initiate actions to achieve goals) and buffer the negative impact of job demands on well-being. This means that the forced remote work of teachers must be fulfilled, and teachers have no choice, which means such a demand weakens motivation. However, a personal resource in the form of passion, which has a primarily motivational dimension, can compensate for the necessity of working from home as well as the workload and feeling of limited organizational support associated with online work.

In the present study, we consider the workload and organizational constraints as job demands, well-being in three markers: emotions, engagement, and job crafting as an outcome, and passion for work as a mediator. Job demands in forced working from home due to the pandemic expressed themselves in the amount of work and the lack of tools as well as procedural and social support at work, so we suppose the perceived workload and organizational constraints are appropriate markers of teacher’s stress. Based on the proposal of Bakker and Demerouti, job resources are perceived as all resources useful at work [26]; therefore, a personal resource is proposed—passion for work. Personal resources refer to people’s beliefs regarding how much control they have over their environment [26]. In the original JD-R theory, personal resources interact with job resources and, as a predictor, explain well-being and other findings [26]. We believe that passion mediates the relation between job demands and engagement or affect and explains the motivational function of job demand (only pro-health) [26,33,37].

Hence, it is derived from the general hypothesis: Work passion mediates between workload, organizational constraints, job crafting, engagement, and affect. Obsessive passion mediates between demands of forced work from home and job crafting, engagement, and affect, whereas harmonious work passion suppresses or mediates these relations. Thus, we expect different patterns of relationships between the perceived demands of work (workload or lack of organizational support at work) and well-being through a passion for work. 

**Figure 1:**
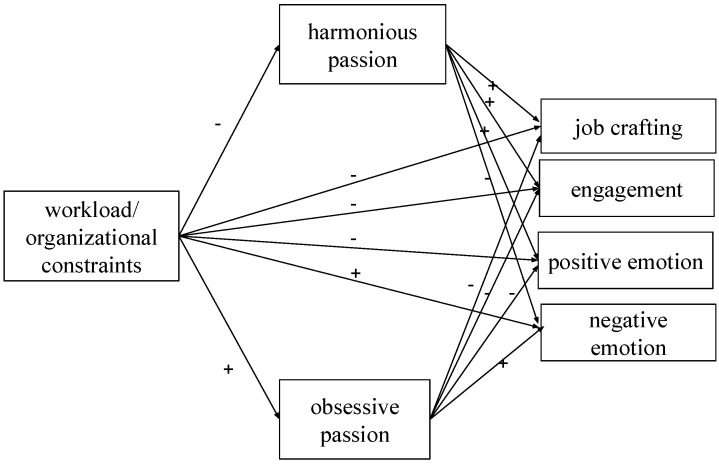
Hypothesized model.

The tested model is presented in Figure 1.

## 2. Materials and Methods

### 2.1. Participants and Procedures

The sample of teachers included 383 people with an average age of 41.93 years (*SD* = 10.24, min = 22, max = 70) with an average professional experience of 16.79 years (*SD* = 11.10, min = 1, max = 40). Women accounted for 95.20% of the sample. Primary school teachers accounted for 57.44% of the sample, kindergarten—11.49%, high school—5.48%, and technical secondary school—4.70%. The rest of the teachers were employed simultaneously in several institutions. During the study, each of the teachers worked remotely.

The survey was conducted online via Google Forms. The link to the survey was shared on social networking sites in the groups of teachers (e.g., Teacher after work; Me, a Teacher). The study was conducted from 8 March 2020 to 19 May 2020.

### 2.2. Measures

The job Crafting Questionnaire [45] in the Polish adaptation by Kasprzak et al. [46] was used to measure job crafting. This questionnaire is used to measure the global job crafting and each of its three areas: tasks (e.g., “Introduce new approaches to improve your work”), interpersonal relations (e.g., “Make an effort to get to know people well at work”) and thinking about work (e.g., “Think about how your job gives your life purpose”). Each of these job crafting dimensions includes five items. The questionnaire consists of 15 statements, which are answered on a scale from 1 to 6 (1—almost never, 6—very often, which means as often as possible). The global result of job crafting is obtained by summing up the results in the subscales. The validity and reliability of the scale have been confirmed in Polish samples [46].

The Utrecht Work Engagement Scale [47] was used to assess engagement to work. This questionnaire consists of nine items and three scales: vigor (e.g., “At my work, I feel strong and vigorous”), dedication (e.g., “I am proud of the work that I do”), and absorption (e.g., “I am immersed in my work”) including three items for each scale, rated on a scale from 0 to 6 (0—never, 6—always). The validity and reliability of the measurement with the indicated version of the scale have been confirmed in research on Polish samples [48].

The Passion Scale [49] in Polish adaptation by Mudło-Głagolska et al. [50] is a tool for assessing harmonious and obsessive passion. It consists of twelve items: 6 each for harmonious and obsessive passion. Individual items have been adapted to the study of work passion, for example: “My work is in harmony with the other activities in my life” (HP) or “I have almost an obsessive feeling for my work” (OP). The answers are given on a 7—point Likert scale from 1 to 7 (1—strongly disagree, 7—strongly agree). The scale obtained satisfactory validity and reliability indicators in Polish samples [50].

The Positive and Negative Experience Scale by Diener et al. [10] in Polish translation by Kaczmarek and Baran [51] is a 12-items tool for studying affective well-being. The scale includes six very general positive and six negative experiences and feelings, thus assessing the full range of positive and negative effects [10]. Each item is rated on a scale from 1 to 5 (1—very rarely or never, 5—very often or always). The tool can calculate positive and negative feelings and the overall affective balance.

The organizational Constraints Scale and the Quantitative Workload Inventory by Spector and Jex [52] in Polish adaptation by Baka and Bazińska [53] were used for cognitive assessment of the work context. The first one contains eleven items (i.e., “How often do you find it difficult or impossible to do your job because of poor equipment or supplies?”), and the second one contains five items (i.e., “How often does your job leave you with little time to get things done?”). Both scales have a 5-point range of responses on a scale of 1 to 5 (1—less than once a month or never, 5—several times a day). The internal consistency of the scales was satisfactory, and the factor structure was confirmed in Polish respondents [53].

In the instructions for individual questionnaires, the respondents were asked to refer to the current period of the COVID-19 pandemic in Poland when answering the questions.

### 2.3. Statistical Analysis

The statistical analysis used JASP version 0.16.0.0 for Windows [54]. A Harman’s one-factor test was performed to diagnose the common method bias [55]. The relationships between the variables were determined based on the r-Pearson coefficient. Structural equation modeling (SEM) was used. JASP mediation analysis is based on the lavaan software. Hypothetical models, considering the intermediary variables in both samples, and their fit were assessed. The maximum likelihood (ML) estimator was used. In the mediation analysis, according to the recommendations of Hayes [56], non-standardized correlation coefficient values are presented in the text and the figures.

## 3. Results

Harman’s one-factor test for common method bias was used to diagnose the potential bias of the common method. The 12.09% of the explained variance proved that the conducted studies were not burdened with a common method bias.

Descriptive statistics and the intercorrelation coefficients are presented in Table 1.

The workload was positively related to organizational constraints and obsessive passion, and poorly with job crafting, and negative emotion. The negative relationship between workload with harmonious passion and positive emotion has been noted. Organizational constraints were positively associated with negative emotion, obsessive passion, and job crafting. A negative relationship was noted with positive emotion, harmonious passion, and engagement. Harmonious passion was positively associated with job crafting, positive emotion, and engagement. A negative relationship with negative emotion has been shown. Obsessive passion was positively associated with job crafting and engagement. Engagement was positively associated with positive emotions and negatively with negative emotions. Positive emotions were negatively related to negative emotions.

Two separate models were used to assess the mediating role of passion for work. In the first model, the independent variable was workload; in the second, it was organizational constraints. The assumption regarding significant relationships between the mediating variable—obsessive passion and positive and negative emotions in both models, as well as with job crafting in the model with the workload as an independent variable (b paths) was not fulfilled; therefore, they were not included in the final models.

Fit indices in models with both workload (model 1: TLI = 0.904, CFI = 0.982, SRMR = 0.038, Chi^2^ = 14.295, *df* = 4, *p* = 0.006 AIC = 5700.664) and organizational constraints (model 2: TLI = 0.999, CFI = 0.999, SRMR = 0.029, Chi^2^ = 8.452, *df* = 2, *p* = 0.038, AIC = 5685.831) show a satisfactory fit to the data in the sample of teachers.

**Figure 2:**
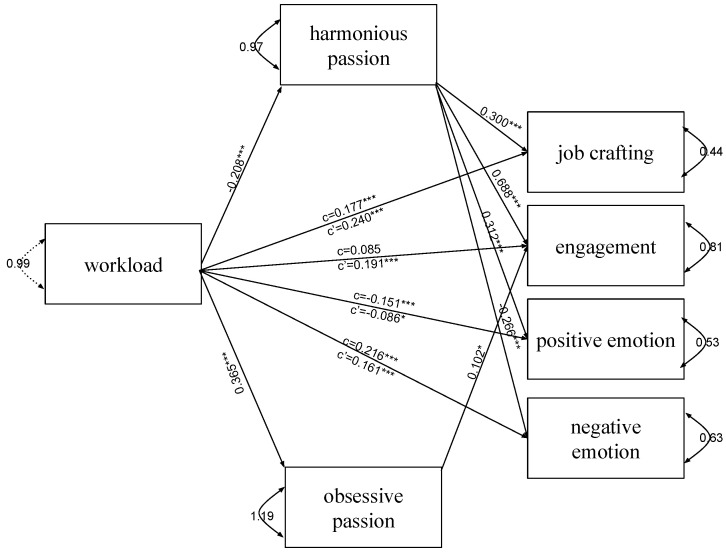
The final model with workload as an independent variable (model 1). The unstandardized path estimates (coefficients) were shown. Note. * *p* < 0.05; *** *p* < 0.001.

**Figure 3:**
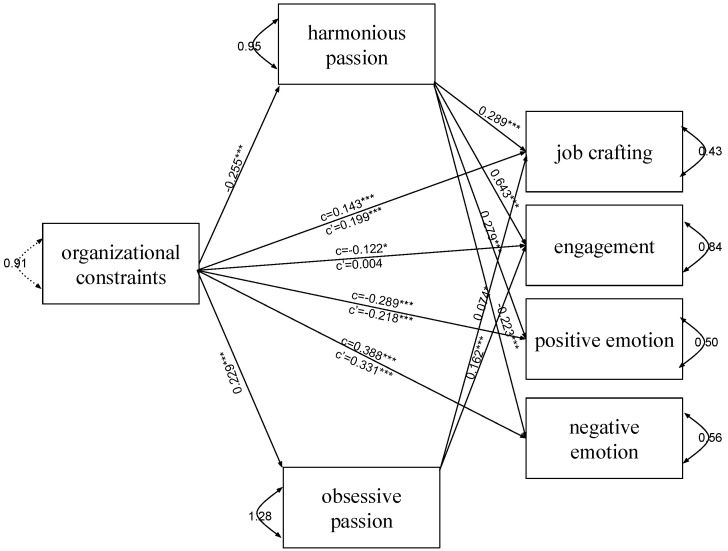
The final model with organizational constraints as an independent variable (model 2). The unstandardized path estimates (coefficients) were shown. Note. * *p* < 0.05; *** *p* < 0.001.

In both models (models 1 and 2), both types of passion mediate the relationship between workload (Figure 2) and organizational constraints (Figure 3) and all indicators of employee well-being—job crafting, engagement, positive emotions, and negative emotions. Harmonious passion was a suppressor or mediator, and obsessive passion mediates the relationship between workload and engagement (*ab*_HP_ = –0.143, 95% = –0.214; –0.074; *ab*_OP_ = 0.037, 95% CI = 0.006; 0.069) (Figure 2) and organizational constraints and job crafting (*ab*_HP_ = –0.074, 95% = –0.108; –0.040; *ab*_OP_ = 0.017, 95% CI = 0.001; 0.033) and engagement (*ab*_HP_ = –0.164, 95% = –0.234; –0.094; *ab*_OP_ = 0.037, 95% CI = 0.010, 0.064) (Figure 3). In mediation, including a third variable, passion, weakens the relationship between the predictor and the dependent variable because the mediator in the causal path between the independent and dependent variables explains some or all of the relationship. In the case of suppression, the inclusion of a harmonious passion strengthens the relationship between the independent variable and the dependent variable. It concerns the mutual suppression of the effects of both predictors, which means that considering only one of them in the analysis may not show its real relationship with the dependent variable [57]. Nevertheless, it can be said that harmonious and obsessive passion together positively affect well-being. The indirect effect of harmonious passion in both models was stronger than obsessive passion (Table 2).

The suppression effect can be indicated in the model of the relationship between workload and organizational constraints and job crafting and engagement. In both models, harmonious passion is a mediator in relation to emotion. Detailed results concerning indirect effects are presented in Table 2.

## 4. Discussion

The results of structural equation modeling (SEM) largely support this model, revealing that work passion could explain employees’ (teachers) well-being. Both models, concerning two different types of demands at work, present suppressive mediation [57]. Work passion intervenes (partially or completely) between work demands—workload and organizational constraints, and behavioral (job crafting), attitudinal (engagement), and affective well-being (positive, negative emotions) of teachers enforced to working from home.

The inclusion of harmonious passion in the model strengthened the positive relationships between workload and organizational constraints and job crafting and weakened the negative relationship with positive emotions and the positive one with negative emotions. The positive relationship between workload and engagement has been strengthened by harmonious passion. The negative relationship between organizational constraints and engagement became positive and weaker.

Obsessive passion mediated workload and engagement and organizational constraints and job crafting, engagement. Positive relationships between variables weakened, e.g., workload and engagement, organizational constraints and job crafting, or strengthened, e.g., organizational constraints and engagement, by an obsessive passion. The indirect role of obsessive passion was relatively low compared to harmonious passion.

### 4.1. Job Demands and Well-Being

Based on the dualistic model of passion, it is expected that both obsessive and harmonious passion boost engagement (attitudinal well-being), job crafting (behavioral well-being), and affective well-being. Work passion manifests itself in the attractiveness of the passion object and the willingness to practice the passion, hence the greater tendency to be engaged and proactive, but the reasons (or circumstances) for engagement are different.

Harmonious passion for work always motivates (conserves energy and directs behavior) regardless of the type of demands because passion reflects the employee’s identity, is stable in time, and is resistant to adverse circumstances, so the employee’s engagement does not weaken or even strengthen. Harmonious passion motivates one to act and even craft a job in order to derive pride and joy from the activity itself or the goal of work, while obsessive passion engages the employee to maintain self-esteem, protect the ego and obtain external benefits [29].

People with obsessive passion invest significant time and effort at work to feel good about themselves and avoid feeling guilty. External benefits (limited while working in isolation), self-protection, and a strong, rigid need to be engaged are unstable and not significant (feeble) motivators. The engagement of these teachers should be weakened than the teachers with harmonious passion. Such an employee engages in work and even crafts work (under the pressure of organizational constraints), but his defensive attitude and rigid form of activation do not allow him to experience positive emotions, which leads to negative emotions (anxiety andnervousness) [31].

Our models support the assumption of the protective role of harmonious work passion against experiencing the negative emotional effects of workload and constraints at work. Harmonious passion pushes to perform work in a flexible way, convenient for the abilities and willingness of the person and therefore acts as an affective well-being stabilizer as well as a cognitive (acquired) attractor of affective and behavioral well-being [58]. The described action of harmonious passion increasing well-being and protecting against stress is consistent with previous studies [11].

Results of previous research about burnout and engagement [37] suggest that forced work at home may be the next new context that prompts teachers to engage and confirm their effectiveness and worth. The search for external or psychological rewards reflects an introjected or identified regulation [30] that manifests in a rigid and maladaptive manner of performing tasks and causes great emotional losses. A rigid form of work does not allow the employee to break away from work, disrupts the balance between work and other activities, and then inhibits the experience of positive emotions and fuels the feeling of guilt and shame during work [29]. The results of this study confirm that passion suppresses the negative effects of demands on motivation at work (engagement and job crafting), and harmonious passion suppresses the costs of demands on emotions.

### 4.2. Types of Demands and Job Crafting and Engagement

Our study also reveals a new perspective on job demands. According to the J-DR proposal, work demands can be challenging and a hindrance depending on its motivational function and the costs or benefits for the employee. This criterium for the division of demands however important, it is not complete [20,59]. We show that one demand in certain working conditions can be perceived as a challenge (e.g., workload), although it is widely recognized as a hindrance. The employee assesses the job demand as challenge or a hindrance not so much in the context of the personal effort put in (or personal costs and benefits), but depending on the circumstances, e.g., the form of work [43]. 

Our models show that the strong protective effect of harmonious passion, despite the impact of obsessive passion, sustained the effect of complete suppression of the relationship between organizational constraints and engagement. Both types of demands predict engagement through harmonious and obsessive passion, but only organizational constraints predict job crafting via both passions.

Perhaps the perception of both demands is different, hence the different sensitivity of these demands to passions. Tasks and time to complete (perform) them relate to the content of the work, defining the employee as a good or an ineffective worker. Workload is the result of coping with tasks, conserving energy, and staying focused on work. Organizational constraints, including lack of tools and procedures and insufficient support from colleagues and supervisors, are external (organizational) elements of the work that are less threatening to the self-concept. In addition, external barriers, however inconvenient, are easy to overcome because they do not take away energy.

In our study, workload causes motivational damage, but under the influence of passion (mainly harmonious), workload loses its negative impact. On the other hand, motivational costs as a result of organizational constraints are smaller because both a controlled and uncontrolled passion operate.

As knowledge workers working with passion and autonomy, previously often with IT technology, teachers perceive organizational constraints as an incentive to engage and craft their work. 

Harmonious passion buffers the negative impact of external constraints of work on engagement and job crafting due to the fullness of energy and the pleasure of being occupied with a favorite activity. Obsessive passion pushes teachers to overcome ineffective procedures, lack of work tools, or resign from tasks in safe circumstances: family support, autonomy, and external demands objectively uncontrolled [22,30,38]. 

Therefore, burdensome work can increase engagement and job crafting when the employees work with passion. The mediating role of both passions is clearer here than between demands and emotional well–being, which were previously revealed in research concerning engagement and burnout [36,37].

To summarise, the indicated mediating mechanism of both types of passion is characteristic of teachers not only due to the specificity of remote work in their activities but also because this professional group is characterized by a significant power of passion. In the study by Carbonneau et al. [60], over 90% of teachers have passion, and in the presented study, the harmonious work passion of teachers is significantly higher than that of the obsessive one (HP: *t* = 10.17, *p* < 0.001, *d* = 0.787; OP: *t* = 4.14, *p* < 0.001, *d* = 0.320).

This mechanism has been identified in the well-being model of teachers working from home, but we believe this mechanism is universal. Passion is a stable tendency to act, bonded with the identity of a person; thus, passion acts regardless of profession and manner of work. Passion is sensitive to different factors, e.g., job resources [61], and causes many positive or negative outcomes [11,31,62]. Mediation of passion has been proved in other work contexts, for example, between demands and engagement and burnout in conventional workplaces, among nurses and teachers [37], between job crafting and burnout and engagement of knowledge workers from Australian and Chinese cultures [36].

### 4.3. Limitations and Future Research

The first limitation of our study is concerned with the cross-sectional design of the study and the characteristics of the sample. Although the tested models support the motivational mediation of work passion in shaping well–being, we propose to use other research procedures that examine causality. In addition, collecting data via the Internet has limited the sample of respondents to people active on social media. The overrepresentation of women and teachers with harmonious passion is the next limitation of the study. The analyzed model did not consider the role of work experience, which should be supplemented in future research. Among teachers, only 15.72% of the respondents were classified as obsessive passionates based on the dominant result [63]. The slight intensity of obsessive passion reduced by the action of the stronger harmonious passion shows that the effect of obsessive passion is weak. Therefore, in future research on the mediating role of obsessive work passion, it is worth trying to include only obsessive passionates with a low result of harmonious passion or to separately analyze the effect of the weak obsessive and harmonious passion and a strong obsessive and harmonious passion. Hence, future research should determine the harmonious and obsessive passion ratio that explains the breakpoint between the adaptive and maladaptive influence of obsessive passion on subjective well–being. Similarly, it seems justified to use latent profile analysis (LPA), which enables the identification of homogeneous subgroups of participants based on common features, in this case, work passion [64].

Secondly, some indirect effects of obsessive passion for work were relatively small. Ho and Astakhova [65] postulate that the relationship between passion and its effects should be treated as curvilinear, according to which a small intensity of obsessive passion does not bring losses, and a small intensity of harmonious passion does not lead to the expected benefits. In future studies devoted to work passion predicting affective, attitudinal, and behavioral well–being, it is necessary to analyze a curvilinear relationship that can explain the dependent variables better than the rectilinear monotone relationship we adopted.

Thirdly, in our study, we asked about the demands of remote work and adopted work resources based on the features of these work environments. In future studies, it is also worthwhile to measure work resources separately as a predictor of employee well-being.

Moreover, the results of the effects of obsessive passion are still unclear. In our study, obsessive passion is positively associated with job crafting, engagement, and emotions, consistent with many previous reports [11] and supports its neutral or even adaptive function.

It is believed that it is worthwhile to examine the proposed models in other socio–sanitary conditions after the end of the COVID-19 pandemic to make sure that the mediating role of passion is indeed a universal mechanism explaining the effects of work demands on well-being in remote and conventionally performed work.

## 5. Conclusions

The structural equation modeling (SEM) confirmed that the work passion, mainly harmonious, is a mechanism explaining the relationship between the demands of forced work from home with teachers’ well-being. Our models support the assumption of the protective role of harmonious work passion against experiencing the negative emotional effects of workload and constraints at work. Moreover, passion for work mediates or suppresses the relationships between these demands and positive emotions, engagement, and job crafting. The direction of impact of harmonious passion and obsessive passion for work is different, but together they have a positive effect on well-being. Harmonious work passionate use the opportunities created by work and have individual resources to adapt to or deal with work demands, which is the starting point for a healthy, unconflicted engagement [37].

Our findings show that work passion is a stronger mediator between organizational constraints and engagement and job crafting than between workload and attitude and behavior due to different perceptions of these demands at work. The studies by Lavigne et al. [33] in the group of teachers emphasized that passion for work influenced employees’ assessment of demands and resources, allowing them to assess the characteristics of work in a way that corresponds to their professional orientation [19], which may protect against negative consequences for the employee.

It is important to maintain teachers’ passion for teaching. School principals should create working conditions for teachers that favor the development of harmonious passion. Teachers should be able to teach the subjects they love and have been trained for while having the necessary resources to meet the demands placed on them.

## Figures and Tables

**Table 1:** Descriptive statistics and scale intercorrelations.

Variable	W	OC	HP	OP	JC	ENG	PE	NE
W	0.89							
OC	0.441 ***	0.91						
HP	–0.207 ***	–0.242 ***	0.84					
OP	0.316 ***	0.190 ***	0.091	0.83				
JC	0.241 ***	0.186 ***	0.344 ***	0.20 ***	0.83			
ENG	0.073	–0.101 *	0.577 ***	0.212 ***	0.369 ***	0.93		
PE	–0.187 ***	–0.343 ***	0.411 ***	–0.040	0.132 *	0.329 ***	0.93	
NE	0.250 ***	0.429 ***	–0.348 ***	0.072	–0.016	–0.179 ***	–0.514 ***	0.88
*M* (*SD*)	3.64 (0.99)	2.43 (0.96)	4.96 (1.01)	3.30 (1.15)	4.26 (0.74)	3.97 (1.15)	3.19 (0.81)	2.82 (0.87)

Note. W—workload, OC—organizational constraints, HP—harmonious passion, OP—obsessive passion, JC—job crafting, ENG—engagement, PE—positive emotion, NE—negative emotion. Cronbach’s alphas are presented in the diagonal. * *p* < 0.05; *** *p* < 0.001.

**Table 2:** Detailed results for indirect effects in the relationship between workload and organizational constraints and well-being indicators—job crafting, engagement, positive and negative emotions, considering the mediation of harmonious and obsessive passion, in subsequent models (1, 2).

Effect	Estimate	SE	*Z*	*p*	*std. est (all)*
Model 1					
W → HP → JC	−0.063	0.017	−3.753	<0.001	−0.085
W → HP → ENG	−0.143	0.036	−3.983	<0.001	−0.126
W → OP → ENG	0.037	0.016	2.320	0.020	0.033
W → HP → PE	−0.065	0.018	−3.698	<0.001	−0.081
W → HP → NE	0.055	0.016	3.482	<0.001	0.064
Model 2					
OC → HP → JC	−0.074	0.017	−4.227	<0.001	−0.096
OC → OP → JC	0.017	0.008	2.099	0.036	0.022
OC → HP → ENG	−0.164	0.036	−4.586	<0.001	−0.138
OC → OP → ENG	0.037	0.014	2.733	0.006	0.031
OC → HP → PE	−0.071	0.017	−4.102	<0.001	−0.084
OC → HP → NE	0.057	0.015	3.701	<0.001	0.063

Note. W—workload, OC—organizational constraints, HP—harmonious passion, OP—obsessive passion, JC—job crafting, ENG—engagement, PE—positive emotion, NE—negative emotion.

## Data Availability

The data presented in this study are available upon request from the corresponding author.
